# Hyperthermic Intraperitoneal Chemotherapy (HIPEC): An Overview of the Molecular and Cellular Mechanisms of Actions and Effects on Epithelial Ovarian Cancers

**DOI:** 10.3390/ijms231710078

**Published:** 2022-09-03

**Authors:** Pei-Qi Lim, I-Hung Han, Kok-Min Seow, Kuo-Hu Chen

**Affiliations:** 1 Department of Medical Education, Taipei Tzu-Chi Hospital, The Buddhist Tzu-Chi Medical Foundation, Taipei 231, Taiwan; 2 Department of Obstetrics and Gynecology, Shin Kong Wu Ho-Su Memorial Hospital, Taipei 111, Taiwan; 3 Department of Obstetrics and Gynecology, National Yang-Ming Chiao-Tung University, Taipei 112, Taiwan; 4 Department of Obstetrics and Gynecology, Taipei Tzu-Chi Hospital, The Buddhist Tzu-Chi Medical Foundation, Taipei 231, Taiwan; 5 School of Medicine, Tzu-Chi University, Hualien 970, Taiwan

**Keywords:** hyperthermic intraperitoneal chemotherapy, HIPEC, ovarian cancer, survival

## Abstract

Most patients with epithelial ovarian cancers (EOCs) are at advanced stages (stage III–IV), for which the recurrence rate is high and the 5-year survival rate is low. The most effective treatment for advanced diseases involves a debulking surgery followed by adjuvant intravenous chemotherapy with carboplatin and paclitaxel. Nevertheless, systemic treatment with intravenous chemotherapeutic agents for peritoneal metastasis appears to be less effective due to the poor blood supply to the peritoneal surface with low drug penetration into tumor nodules. Based on this reason, hyperthermic intraperitoneal chemotherapy (HIPEC) emerges as a new therapeutic alternative. By convection and diffusion, the hyperthermic chemotherapeutic agents can directly contact intraperitoneal tumors and produce cytotoxicity. In a two-compartment model, the peritoneal–plasma barrier blocks the leakage of chemotherapeutic agents from peritoneal cavity and tumor tissues to local vessels, thus maintaining a higher concentration of chemotherapeutic agents within the tumor tissues to facilitate tumor apoptosis and a lower concentration of chemotherapeutic agents within the local vessels to decrease systemic toxicity. In this review, we discuss the molecular and cellular mechanisms of HIPEC actions and the effects on EOCs, including the progression-free survival (PFS), disease-free survival (DFS) and overall survival (OS). For primary advanced ovarian cancers, more studies are agreeing that patients undergoing HIPEC have better surgical and clinical (PFS; OS) outcomes than those not, although one study reported no differences in the PFS and OS. For recurrent ovarian cancers, studies have revealed better DFS and OS in patients undergoing HIPEC than those in patients not undergoing HIPEC, although one study reported no differences in the PFS. HIPEC appears comparable to traditional intravenous chemotherapy in treating advanced EOCs. Overall, HIPEC has demonstrated some therapeutic benefits in many randomized phase III trials when combined with the standard cytoreductive surgeries for advanced EOCs. Nevertheless, many unknown aspects of HIPEC, including detailed mechanisms of actions, along with the effectiveness and safety for the treatment of EOCs, warrant further investigation.

## 1. Introduction

Ovarian cancer is the second most common gynecologic malignancy in developed countries and the third most common gynecologic malignancy in developing countries [1]. Approximately 75% of affected women have stage III (disease that has spread throughout the peritoneal cavity or that involves lymph nodes) or stage IV (disease that has spread to more distant sites) disease at diagnosis. The 10-year survival rate of women with advanced disease is only 10% to 15%, and it has been the same for the past 20 years [2]. The most effective treatment for advanced diseases involves a cytoreductive debulking surgery to reduce the tumor burden followed by six cycles of adjuvant intravenous chemotherapy with carboplatin and paclitaxel. Alternatively, an interval cytoreductive surgery is performed after three cycles of neoadjuvant chemotherapy [3].

Peritoneal carcinomatosis of ovarian, fallopian tube or primary peritoneal cancers is deemed a technical obstacle to complete resection [4]. However, systemic treatment with intravenous chemotherapeutic agents for peritoneal metastasis appears to be less effective as compared to its use for lung or liver metastases. This realistic finding is due to the poor blood supply to the peritoneal surface with low drug penetration into tumor nodules, thereby preventing eradication of tumor growth. Based on this reason, locoregional drug delivery has emerged, and this route allows chemotherapeutic agents to be administered in a higher dose by instillation in the peritoneum (intraperitoneal) [5,6]. Intraperitoneal administration of chemotherapeutic agents specifically targets remaining microscopic diseases after complete cytoreduction. By way of delivering chemotherapy directly within the peritoneal cavity, poorly vascularized tumors could be more exposed to the local high concentration of chemotherapy. On the other hand, the blood–peritoneal barrier also limits the passage of this high concentration of chemotherapy back to the blood vessels, thus minimizing systemic toxicity while maximizing local effects. Currently, many randomized trials, meta-analyses and real-world data reveal that the administration of adjuvant intraperitoneal chemotherapy after cytoreduction improves overall survival (OS) and progression-free survival (PFS) in patients with advanced ovarian cancers [2].

Hyperthermic intraperitoneal chemotherapy (HIPEC) was first used clinically in 1980 by Spratt et al., who performed hyperthermic chemoperfusion with thiotepa in a patient with pseudomyxoma peritonei. Theoretically, hyperthermia has direct cytotoxic effects on tumor cells and induces the production of heat shock proteins that serve as receptors for natural killer (NK) cells; these actions lead to apoptosis of tumor cells and inhibition of angiogenesis. Practically, heat is directly cytotoxic, improves chemotherapy penetration into the tumor tissues and is synergistic with commonly used chemotherapeutic agents including cisplatin, paclitaxel, oxaliplatin and mitomycin c [2,7]. The optimal temperatures for administration of chemotherapeutic agents fall between 42 and 43 °C. The synergy between heat and drug cytotoxicity starts at 39 °C and falls off at 43 °C. Temperatures above 44 °C can cause apoptosis in normal cells [6].

In this review, we discuss the underlying molecular and cellular mechanisms of actions from HIPEC and chemotherapeutic agents, and the results from the clinical studies that investigated the effects of HIPEC on epithelial ovarian cancers (EOCs).

## 2. Literature Review: The Database, Searching Terms and Strategies

**Figure 1:**
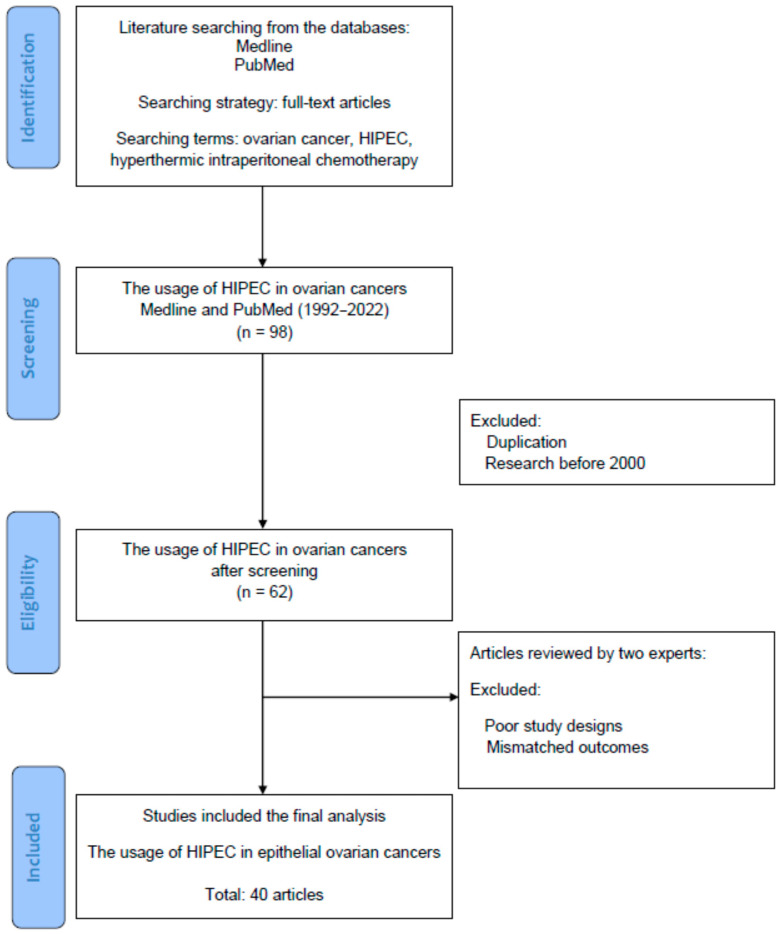
The diagram of article selection, screening and inclusion from the literature.

Figure 1 is a flowchart of the searching, screening, and including process of the references we selected from the literature. In this review, all of the reference articles were retrieved from the databases Medline and PubMed using the searching terms “ovarian cancer”, “hyperthermic intraperitoneal chemotherapy”, or “HIPEC” for the research topic. In this (identification) stage, only full-text articles were considered for a further analysis. Up to 31 March 2022, we searched potential articles in the literature from the databases Medline and PubMed. The literature published from 1 January 1992 to 31 March 2022 was searched to identify eligible articles for the review. In the second (screening) stage, duplicated articles and articles published prior to 2000 were excluded to ensure the novelty of this review. In the next stage, two experts in the field independently inspected the contents of articles including demographics, research designs and outcomes and identified potentially eligible studies for exclusion and inclusion. The retrieved articles with poor research designs or mismatched outcomes were excluded at this stage. The discrepancies between the two experts were discussed by their mutual communication to reach a consensus. All eligible articles using the searching terms and strategies (database searching, screening, selection and inclusion of eligible articles) were included in the review. From a total of 98 articles identified in the searching process, 40 articles were ultimately collected for review.

## 3. Molecular and Cellular Mechanisms of Actions: HIPEC and Chemotherapeutic Agents

### 3.1. HIPEC

The first study for HIPEC, which started with an animal model, was conducted by Euler in 1974. Because traditional treatment of surgical resection or systemic intravenous chemotherapy may be less effective, the goal of hyperthermia is to enhance the antitumor effect of chemotherapeutic agents. It is very important to understand the pharmacokinetic behavior and drug–tissue transport after intraperitoneal chemotherapy. However, both standardization of the HIPEC techniques and the results from clinical trials are still equivocal. Therefore, it is still controversial with regard to the efficacy and safety of HIPEC for clinical malignancies including ovarian, colorectal, appendix and peritoneal cancers. The clinicians in favor of HIPEC use RCTs of ovarian cancers to support the benefit of HIPEC, while those who oppose the application of HIPEC use negative RCT results of colorectal cancers [5].

During HIPEC, relevant drug properties of chemotherapeutic agents include molecular weight, hydrodynamic size, charge and configuration; relevant physical parameters, treatment variables and carrier properties include hydrostatic pressure, temperature and viscosity. An overview of the drug properties, physical parameters, treatment variables, carrier properties and tumor microenvironment (TME) properties is summarized in Table 1.

The relevant molecular and cellular mechanisms of HIPEC actions are described below. Two critical factors that affect tissue transport after intraperitoneal drug delivery (IPDD) are convection (pressure gradient) and diffusion (concentration gradient) [5].

**Figure 2:**
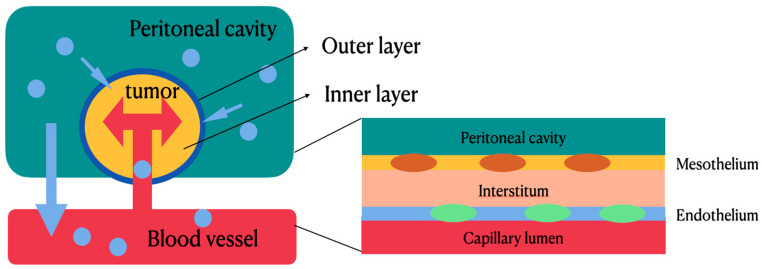
Bidirectional models for intravenous and intraperitoneal therapy. The outer layer of the tumor can achieve a high concentration of drug levels by direct exposure, while the drug can reach the inner core layer of the tumor by microcirculation through systemic circulation. Between the two compartments is the peritoneal–plasma barrier, which can decrease drug clearance rate from peritoneal cavity to systemic circulation.

Figure 2 shows the pharmacokinetic model of HIPEC which is delivered based on (Cp/Cb)IP/(Cp/Cb)IV, where Cp represents the concentration of the drug in the peritoneum and Cb represents the concentration of the drug in blood. In terms of chemotherapeutic agents used during HIPEC, area under the curve (AUC) represents drug concentration and exposure time to the tumor in the peritoneum [8,9]. The mechanisms of HIPEC involve a two-compartment model to describe the pharmacokinetics of IPDD [5,8,9,10]; intraperitoneal chemotherapy drugs are delivered via convection and diffusion gradients. Figure 2 illustrates the bidirectional models for intravenous (IV) and intraperitoneal (IP) therapy. The outer layer of the tumor can achieve a high concentration of drug levels by direct exposure (IP), while the drug can reach the inner core layer of the tumor by microcirculation through systemic circulation (IV). Between the two compartments is the peritoneal–plasma barrier (PPB), which plays a key role in the compartmental model and can decrease the drug clearance rate from the peritoneal cavity to systemic circulation. During IP therapy, the PPB blocks the leakage of chemotherapeutic agents from the peritoneal cavity and tumor tissues to local vessels, thus maintaining a higher concentration of chemotherapeutic agents within the tumor tissues to facilitate tumor apoptosis, and a lower concentration of chemotherapeutic agents within the local vessels to decrease systemic toxicity.

The molecular and cellular actions of HIPEC are mainly via convection, diffusion and hyperthermia.

#### 3.1.1. Convection

Convection is used to describe the process by which heat is transferred by movement of a heated fluid such as air or water. Convection of drug transport is proportional to the difference in pressure of fluid filling the peritoneal cavity and pressure of the tumor stromal tissues. As described above, the chemotherapeutic effect is associated with the cellular and stromal structure. The hydrostatic pressure refers to the intraperitoneal fluid exert pressure, which is about 10–20 cm H_2_O. Regarding the tumor tissue pressure, preclinical experiments and numerical simulation have suggested that the pressure of the tumor tissues is much higher than that of the normal tissues [1]. There are three reasons for the observed results. First, the tumor tissues have elevated interstitial fluid pressure due to elevated blood glow, leaky capillaries and deficient lymphatic drainage. Second, the surrounding solid tissue stress would increase as tumors grow. Third, the residual solid stress would be stored as elastic energy. As the tumor is cut, bulging and expansion could be observed. There are also some factors that affect pressure-driven (convective) drug transport. According to hydraulic conductivity of the tissue, factors such as viscosity of interstitial fluid and stiffness of the tumor stroma would affect the convention [5].

#### 3.1.2. Diffusion

The diffusion mechanism of the drugs can be described as the Fick law, which explains that diffusive mass transport is driven by a concentration gradient. In addition, the rate of diffusion is also affected by temperature, physicochemical drug properties and stromal architecture [5]. Associated drug properties include molecular weight, hydrodynamic size, charge and configuration, while stroma and excellular matrix (ECM) properties include cellular composition, density, stiffness, visco-elasticity and geometrical fiber arrangement [5]. The tumor tissues have some characteristics that would increase its stiffness. For example, the cancer tissues have elevated collagen I, resulting in higher stiffness compared with the normal tissues. Additionally, cross-linking enzymes in the tumors would produce more lysyl oxidase (LOX) compared with those in the normal tissue, resulting in more stromal stiffness. Geometric arrangement of collagen fibers would affect drug effusion as well. Fibers arranged tangentially from the tumor surface would direct drug effusion away from the tumor, while fibers radially aligned would have more drug effusion.

#### 3.1.3. Hyperthermia

In the 18th century, doctors noted that tumors would shrink when patients had febrile diseases. Thus, hyperthermia was studied and used to treat different types of cancers. Hyperthermia has been researched for decades and is mostly used in combination with chemotherapy, radiotherapy, or immunotherapy.

The mechanism of hyperthermia in treating cancers is established based on different vascular structures between normal tissues and tumor tissues. In normal tissues, the vessels comprise a network of arterioles, capillaries and veins; in tumor tissues, the vessels are arranged chaotically, and there is a lack of smooth muscle and innervation [11]. Studies have shown that when regional temperature exceeds 42 °C, tumors are damaged directly due to increased permeability. Moreover, fluid and protein would accumulate in the microenvironment because of increased permeability, causing an increase in the interstitial fluid pressure, which can compress the vessels and decrease vascular perfusion in the tumors [11].

Hyperthermia can also increase the fluidity of the bilayer of phospholipids in tumor cells. Therefore, membrane permeability would increase, and viscosity of the membrane would decrease, resulting in an increased cellular uptake of drugs. On the other hand, studies have shown the synergistic effect of hyperthermia and cisplatin (cPt) at 43 °C [11]. Hyperthermia can likely modulate the function of cPt transporter Ctr1, resulting in increased cPt uptake and enhanced cytotoxicity [9]. In clinical practice, the usage of chemotherapy combined with hyperthermia is employed in HIPEC and hyperthermic intravesical chemotherapy (HIVEC).

### 3.2. Paclitaxel

There is compelling evidence proving that paclitaxel (PTX) can kill cancer cells through induction of apoptosis. This is predominantly based on paclitaxel’s combination with microtubules to affect microtubule stabilization. As a result, consequent arrest of the cell cycle at the mitotic phase can be determined as paclitaxel-induced cytotoxicity [12].

**Figure 3:**
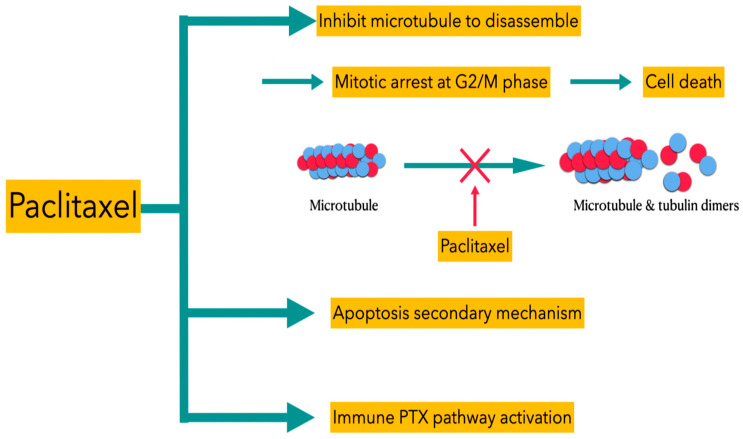
Different types of PTX pathways for inducing death of tumor cells.

There are also studies suggestive of different sensitivities of paclitaxel to microtubules of different statuses, and the concentration of paclitaxel is viewed as the main factor of the apoptogenic mechanism [12,13]. The detailed mechanisms of paclitaxel’s actions can be described as three types of pathways (Figure 3). First, paclitaxel inhibits the microtubules to disassemble to form tubulin dimers, thus blocking the growth of the tumors at the G2/M phase to induce subsequent cell death. Second, apoptosis of tumor cells is also facilitated by paclitaxel via the p53 and *reactive oxygen species* (ROS) pathway. Finally, paclitaxel can induce immune PXT pathway activation to inhibit the growth of the tumor cells. In comparison to other chemotherapeutic agents, paclitaxel is a water-insoluble and high-molecular-weight compound, so intraperitoneally (IP) administered paclitaxel is gradually drained from the peritoneum through lymphatic stomata. The characteristic of prolonged retention for IP paclitaxel allows it to directly and progressively penetrate peritoneal disseminated tumors.

An improved understanding of the cell cycle and apoptosis is helpful in depicting paclitaxel-induced apoptosis. Based on the aforementioned pathways, novel paclitaxel-based regimens can emerge for next-generation cancer therapy.

### 3.3. Cisplatin

Platinum-based anticancer drugs are widely used in chemotherapy to treat neoplasms by affecting DNA and subsequent RNA transcription and translation. Cisplatin is one of the most widely used platinum-based chemotherapy agents used to attack different cancers and sarcomas. The main mechanism of cisplatin is that it cross-links with the purine bases on DNA, leading to impairment in DNA repair and subsequent cell death [14]. Furthermore, cisplatin can also attack mitochondria and trigger the production of ROS. These actions can destroy lysosomes, causing a release of lysosomal protease, and impair the endoplasmic reticulum to affect calcium storage [15]. The main obstacle in using this type of drug is the development of drug resistance and toxicity. It is important to understand the mechanisms of action of drug transportation and metabolic pathways. Much evidence has indicated that the therapeutic and toxic effects of platinum drugs on cells are not only due to covalent adduct formation between platinum complexes and DNA, but also RNA and many proteins. Some studies have suggested that drug resistance of platinum-based chemotherapeutic agents is mainly induced by increasing expression of various transporters and increasing repair of platinum–DNA adducts. In terms of precision medicine, functional genomics is important to predict the platinum–drug response of patients, and genetic polymorphism constitutes the basis of individualized cancer therapy [15].

## 4. Therapeutic Effects of HIPEC in Epithelial Ovarian Cancers

The therapeutic effects of HIPEC on women with ovarian cancers have been explored. Table 2 and Table 3 are summaries of clinical studies that focused on HIPEC treatment for primary advanced ovarian cancers and recurrent ovarian cancers, respectively.

### 4.1. Primary Advanced Ovarian Cancers

OVHIPEC, an open-label, multicenter, randomized phase 3 trial in patients with stage III ovarian cancer who were not eligible for primary cytoreductive surgery (CRS), was conducted by W.J van Driel et al. A total of 245 patients who had at least stable disease after three cycles of carboplatin (AUC of 5 to 6 mg per milliliter per minute) and paclitaxel (175 mg per square meter of body-surface area) underwent interval cytoreductive surgeries either with or without administration of HIPEC with cisplatin (100 mg per square meter). Three additional cycles of carboplatin and paclitaxel were administered postoperatively. In this trial, 89% of the patients in the interval CRS-only group and 81% of the patients in the interval CRS plus HIPEC group experienced an event of disease recurrence or death (hazard ratio (HR), 0.66; 95% confidence interval (CI), 0.50–0.87; *p* = 0.003) after a median follow up of 4.7 years. In this trial, HIPEC added to CRS increased the median PFS by 3.5 months. The median OS of pateints with ovarian cancers was 33.9 for the interval CRS group and 45.7 months for the interval CRS plus HIPEC group [2].

A meta-analysis of 13 comparative studies including randomized controlled trials and case–control trials showed better outcomes after treatment with surgeries and HIPEC in patients with primary advanced ovarian cancers (pooled HR for OS, 0.54; 95% CI, 0.45–0.66; pooled HR for PFS, 0.45; 95% CI, 0.32–0.62). HIPEC also led to favorable clinical outcomes (HR: 0.64, 95% CI: 0.50–0.82; HR: 0.36, 95% CI: 0.20–0.65) for stage III and IV ovarian cancers with initial diagnosis [16]. Another randomized controlled trial which had been conducted by Lim MC et al. enrolled 184 patients with stage III and IV ovarian cancers and randomly allocated them to the trial arm (HIPEC, cisplatin 75 mg/m^2^, 90 min) and control arm (no HIPEC), intraoperatively based on residual tumor (tumor size < 1 cm). In the HIPEC and control groups, the 2-year PFS was 43.2% and 43.5%, and the 5-year PFS was 20.9% and 16.0%, respectively (*p* = 0.569). The 5-year OS was 51.0% and 49.4% in the HIPEC and control group, respectively (*p* = 0.574). The survival analysis did not show the statistical difference between the HIPEC and control arms [17]. However, a recent multicenter retrospective cohort study of 584 women who underwent up-front surgery for stage III ovarian cancers showed significantly improved survival in the HIPEC arm (49.8 months vs. 34.0 months; HR: 0.63, 95% CI: 0.49–0.82, *p* < 0.001). The 3-year overall survival rate was 60.3% (95% CI: 55.3–65.0%) for patients undergoing primary cytorecductive surgery (PCS) plus HIPEC and 49.5% (95% CI: 41.0–57.4%) for patients undergoing PCS alone (weighted HR: 0.64; 95% CI: 0.50–0.82; *p* < 0.001). Complete PCS with HIPEC was associated with the best survival outcomes, with a median OS of 53.9 months and a 3-year OS rate of 65.9% [18,19].

### 4.2. Recurrent Ovarian Cancers

In an 8-year period (2006–2013) of a prospective randomized phase III study, 120 women with advanced ovarian cancers (International Federation of Gynecology and Obstetrics (FIGO) IIIc and IV) who experienced disease recurrence after initial treatment with conservative or debulking surgery and systemic chemotherapy were randomized into two groups. Group A comprised 60 patients treated with CRS followed by HIPEC and then systemic chemotherapy. Group B comprised 60 patients treated with CRS only and systemic chemotherapy. In group A patients, CRS was followed by the administration of HIPEC and subsequent systemic chemotherapy. The HIPEC protocols used were as follows: for platinum-sensitive disease (*n* = 34): cisplatin 100 mg/m^2^ and paclitaxel 175 mg/m^2^ delivered for 60 min at 42.5 °C; for platinum-resistant disease (*n* = 26): doxorubicin 35 mg/m^2^ and paclitaxel 175 mg/m^2^ (or mitomycin 15 mg/m^2^) delivered for 60 min at 42.5 °C. Another group of patients (group B) underwent CRS followed by systemic chemotherapy. The mean survival was 26.7 months in group A versus 13.4 months in group B (*p* < 0.006). The 3-year survival was 75% in group A versus 18 % in group B (*p* < 0.01). Further analyses revealed that complete cytoreduction was associated with a longer survival [20].

In another descriptive study reported by Cascales-Campos in 2011, the outcomes of both primary (*n* = 35) and recurrent (*n* = 11) stage IIIc ovarian cancer patients (*n* = 46) who underwent peritonectomy with HIPEC were analyzed postoperatively. The procedures were carried out through the fast-track program, which used a comprehensive approach designed to accelerate recovery, reduce morbidity and shorten convalescence to ultimately improve outcomes and reduce costs. A total of 37 patients (80.4%) received systemic chemotherapy (3–18 cycles per patient) before HIPEC and surgery. The median operative time was 380 min. CC-0 (no macroscopic tumor residue at the end of cytoreduction) was achieved in 38 patients (82.6%), and CC-1 (less than 2.5 mm of tumor residue after cytoreduction) in the remaining 8. The major morbidity rates were 15.3%, and the most common complication was paralytic ileus. There was no mortality related to the procedure, and the mean postoperative stay was 6.9 days (3–11 days). The study concluded that peritonectomy procedures with HIPEC in advanced ovarian carcinoma was possible under fast-track surgery programs in patients with low-volume peritoneal carcinomatosis [21].

In a randomized phase II trial published in 2021, patients with recurrent EOCs were intraoperatively randomly assigned to either additional carboplatin HIPEC and cytoreductive surgeries or cytoreductive surgeries only, followed by five or six cycles of postoperative IV carboplatin-based chemotherapy, respectively. In a total of 98 patients, 49 (50%) received HIPEC. The median PFS in the HIPEC and standard arms was 12.3 and 15.7 months, respectively. Likewise, there was no significant difference in the median OS (52.5 months vs. 59.7 months) [22]. In contrast, Zhang G et al. showed that HIPEC was associated with improved OS (HR: 0.45, 95% CI: 0.24 to 0.83). However, no difference in the PFS was observed between the HIPEC group and the non-HIPEC group either (HR: 0.55, 95% CI: 0.27 to 1.11) [16].

## 5. The Safety, Adverse Effects and Quality of Life in Patients Who Undergo HIPEC

Based on a review of the literature from 2008 to 2014, the morbidity and mortality from HIPEC was thought to be higher than that from CRS alone [23]. However, adverse events from grade 3 to 5 in the OVHIPEC trial were reported in 30 patients (25%) in the interval CRS group and in 32 patients (27%) in the interval CRS and HIPEC group (*p* = 0.76). The incidence of adverse events was not statistically different between these two arms. In terms of side effects of HIPEC, the most common grade 3 to 4 events were abdominal pain, infections, ileus, thromboembolic events and pulmonary events [2,3]. Another prospective, randomized multicenter trial reported in 2017 also showed no differences between the postoperative outcomes, including extent of surgery, estimated blood loss, residual tumor and hospitalization day between both groups, except operation time (487 min. vs. 404 min., *p* < 0.001) due to the HIPEC procedure. The most common adverse event was anemia: 67.4% in the HIPEC group and 50% in the control group (*p* = 0.025). The other common toxicity in the HIPEC group was the elevation of creatinine (15.2% vs. 4.3%, *p* = 0.026). There were no differences between groups in the incidence of transfusion (35.9% vs. 29.3%, *p* = 0.432), neutropenia (19.6% vs. 10.9%, *p* = 0.151) and thrombocytopenia (9.8% vs. 3.3%, *p* = 0.136) [17].

Acute renal failure is one of the most common toxicities of cisplatin. Cisplatin-related renal toxicity appears to be preventable by administration of sodium thiosulfate to protect renal function [24]. A retrospective study showed that the widespread use of RIFLE criteria for acute renal dysfunction would have major benefits in terms of accurately diagnosing patients undergoing HIPEC procedures [25]. Volume status optimization, early nutritional support, sufficient anticoagulation and point-of-care coagulation management are also encouraged postoperatively after the CRS and HIPEC procedures [26].

The effect of HIPEC on the patient’s health-related quality of life (HRQoL) was evaluated in the OVHIPEC trial. The researchers concluded that the addition of HIPEC to interval CRS does not negatively impact HRQoL in patients with stage III ovarian cancers [27]. Currently, there is still no conclusion nor consensus regarding the usage regimen and temperature setting of HIPEC yet. Because the effectiveness and adverse events are greatly affected by the time of administration, more clinical trials for the optimization and establishment of HIPEC are required in the future [28].

The main risk factors for prolonged length of stay after CRS/HIPEC were advanced age, hypoalbuminemia and multivisceral resection [29]. A retrospective single-center review in April 2021 presented a comparative analysis of the outcomes of CRS and HIPEC between patients under 65 and those ≥65 years. A total of 245 patients underwent CRS and HIPEC during the study period, with 76/245 (31%) ≥ 65 years at the time of intervention. The median length of hospital stay in the ≥65-year-old group was 14.5 days vs. 13 days in the <65-year-old group (*p* = 0.01). Likewise, significant morbidity (Clavien–Dindo ≥ Grade IIIa) was higher in the ≥65-year-old group than in the <65-year-old group (18.4% vs. 11.2%). This study demonstrated a higher perioperative major morbidity in the ≥65-year-old group, but a lower mortality in the patients undergoing CRS/HIPEC for disseminated intraperitoneal malignancy [30].

However, the effects and adverse effects of HIPEC remain to be investigated due to the relatively small sample size of the existent studies.

## 6. Results and Discussion

There are already several studies investigating the underlying molecular and cellular mechanisms and therapeutic effects of HIPEC. Among these studies, AUC, IPDD and other variables have been considered for the better efficacy of HIPEC and mitigation of adverse effects. Herein, the model of the two-compartment theory is demonstrated to explain the actions of HIPEC. Because of higher local exposure to therapeutic drugs for intraperitoneal tumors and lower systemic toxicity due to decreased drug concentration in systemic circulation, HIPEC has become more popular nowadays. For primary advanced ovarian cancers, more studies are in agreement that patients undergoing HIPEC have better surgical and clinical (PFS; OS) outcomes than those not, although one study reported no differences in the PFS and OS. For recurrent ovarian cancers, studies have revealed a better DFS and OS in patients undergoing HIPEC than those in patients not undergoing HIPEC, although one study reported no differences in the PFS. HIPEC appears to be comparable to traditional intravenous chemotherapy in treating advanced EOCs. However, there are still limitations for the usage of HIPEC in clinical practice. First of all, the ideal temperature for HIPEC actions remains unclear [5]. An improved understanding of cell cycles, thermo-effects and paclitaxel-induced apoptosis could help clinicians find better temperature and paclitaxel-based regimens for cancer therapy. Second, cisplatin has a synergistic effect with hyperthermia, and the primary mechanism of HIPEC is accelerated platination of DNA to lead to cell death. However, drug resistance and toxicity are two major obstacles of intraperitoneal cisplatin, and further surveys for drug resistance and toxicity are suggested. Furthermore, HIPEC may be associated with a potentially increased risk for platinum-refractory or -resistant diseases. Finally, higher surgical complexity during the HIPEC procedure may contribute to elevated complication rates without improving oncologic outcomes in patients who undergo intraperitoneal chemotherapy [31].

For women with advanced ovarian cancers who are eligible for up-front surgery, the efficacy of HIPEC has yet to be shown in a prospective RCT. International groups agreed that these women should only receive HIPEC within a clinical trial. The OVHIPEC-2 study, an international multicenter RCT of HIPEC at the time of up-front surgery for stage III ovarian cancer was then started. The primary objective of the study was to evaluate the effect of HIPEC on the OS in patients with FIGO stage III epithelial ovarian cancers who were treated with primary cytoreductive surgery resulting in no residual disease, or residual disease up to a maximum dimension of 2.5 mm [18,32,33]. However, there were criticisms of OVHIPEC questioning the use of HIPEC. Some of the significant criticisms include: (1) long accrual time (9 years) with the trial being performed in the era of anti-angiogenesis therapy and poly (adenosine diphosphate ribose) polymerase (PARP) inhibitors, (2) lack of stratification (e.g., BRCA status, histology), (3) small study size, leading to a difference of 15 deaths between groups and (4) use of the RFS as the primary endpoint, while the OS was a secondary endpoint [34,35,36,37]. The randomized phase III trial in recurrent EOC by Spiliotis et al. was also criticized for its study design. This study did not report the PFS, postoperative complication rate or adjuvant chemotherapies [34].

In the recurrent setting, no consensus exists on the protocol to be used. This includes variation in the choice of intraperitoneal chemotherapy drugs, length of time, timing of surgery, open-versus-closed technique and optimal temperature [7]. Similarly, different results have been reached in past studies that investigated the usage of HIPEC for primary or recurrent advanced ovarian cancers. In practice, several discrepancies in results and conclusions among these studies may originate from the clinical setting: the including criteria, HIPEC temperature, dosage of intraperitoneal drugs and duration of use.

The cost-effectiveness of HIPEC was studied, and it was found that the addition of HIPEC with cisplatin at the time of interval cytoreduction following neoadjuvant chemotherapy is cost-effective compared to a primary debulking surgery and adjuvant chemotherapy [38]. On the basis of the trial data of the OVHIPEC, the treatment with interval CRS and HIPEC in patients with stage III ovarian cancers was accompanied by a substantial gain in quality-adjusted life years [39]. From the viewpoint of health economics, the usage of HIPEC is worth considering for the treatment of advanced EOCs.

For the staff involved in the procedure, protocols are needed for the introduction, handling and management of chemotherapeutic agents in the operating room to minimize the HIPEC risk. Individual exposure during CRS and HIPEC may arise from different routes, such as air contamination, direct contact, manipulation of perfusates or chemotherapy solutions and manipulation of objects/tissues exposed to chemotherapeutics. Guidelines for safe administration of HIPEC, including environmental contamination risk management, personal protective equipment and occupational health issues, are yet to be established [40].

The molecular and cellular mechanisms of EOCs and their treatment remain not fully understood. Further efforts are still required to investigate the therapeutic role of HIPEC in ovarian cancers. To minimize the heterogeneity of research in the future, standardization of several important factors in EOCs and relevant treatment should be considered. Critical factors include the HIPEC temperature, dosage of intraperitoneal drugs and duration of use, all of which have significant impacts on the therapeutic effects. Furthermore, the severity and outcomes in the patients with advanced EOCs also need standardization. Moreover, a larger sample size is required to obtain a reliable conclusion and to improve the reproducibility of the research results.

## 7. Conclusions

The molecular and cellular actions of HIPEC are mainly achieved via convection, diffusion and hyperthermia. The model of the two-compartment theory is demonstrated to explain the actions of HIPEC. Because of higher local exposure to therapeutic drugs for intraperitoneal tumors and lower systemic toxicity due to decreased drug concentration in systemic circulation, HIPEC has become an alternative in the adjuvant or neoadjuvant treatment of EOCs. For primary advanced ovarian cancers, more studies agree that patients undergoing HIPEC have better surgical and clinical (PFS; OS) outcomes than those not, although one study reported no differences in the PFS and OS. For recurrent ovarian cancers, studies have revealed better DFS and OS in patients undergoing HIPEC than those in patients not undergoing HIPEC, although one study reported no differences in the PFS. HIPEC appears comparable to traditional intravenous chemotherapy in treating advanced EOCs. However, there are still some limitations for the usage of HIPEC in clinical practice.

Overall, HIPEC has demonstrated some therapeutic benefits in many randomized phase III trials when combined with the standard CRS for advanced EOCs. This review could help healthcare practitioners understand the recent evidence regarding the usage of HIPEC for advanced EOCs and suggests future developments in this emerging area. Nevertheless, many unknown aspects of HIPEC, including detailed mechanisms of actions, along with its effectiveness and safety for the treatment of epithelial ovarian cancers, warrant further investigation.

## Figures and Tables

**Table 1:** An overview of relevant factors that affect tissue transport during intraperitoneal drug delivery (IPDD).

Physical parameter, treatment, variable, carrier properties	
Hydrostatic pressure	Osmolarity
Temperature	pH
Viscosity	Exposure time
Drug properties	
Concentration Molecular weight Hydrodynamic diameter	Configuration Water solubility Protein binding Charge ionization
TME properties	
Interstitial fluid pressure	Retardation coefficient
Solid pressure	Cellular composition
Hydraulic conductivity	Stromal and vascular density
Viscoelasticity, stiffness	Geometrical arrangement

**Table 2:** A summary of clinical studies investigating HIPEC treatment for primary advanced ovarian cancers.

Authors	Study Design	Patients	Treatment	Results
Lim MC (2017)	Prospective, randomized multicenter trial	1. Patients with stage III/IV primary advanced epithelial ovarian cancer who have optimal cytoreductive surgery; 2. Total: 184 patients.	Groups: 1. Intraoperative HIPEC with cisplatin (75 mg/m^2^, 90 min); 2. Control arm (no HIPEC)	1. HIPEC: 2-year PFS: 43.2%; 5-year PFS: 20.9%; 5-year OS: 51.0%. 2. Control group: 2-year PFS: 43.5% 5-year PFS: 16.0% (*p* = 0.569) 5-year OS: 49.4% (*p* = 0.574)
W.J. van Driel (2018)	Multicenter, prospective, randomized phase III trial	1. Newly diagnosed stage III epithelial ovarian, fallopian tube, or peritoneal cancers; 2. Not eligible for primary cytoreductive surgery; 3. 245 patients.	Three cycles of carboplatin (AUC of 5 to 6 mg/mL/min) and paclitaxel (175 mg per square meter of body-surface area) after interval cytoreductive surgery for all patient. Groups: 1. HIPEC with cisplatin (100 mg per square meter), with intra-abdominal temperature of 40 °C (104 °F), open technique; 2. Without HIPEC.	1. Median follow up: 4.7 years 2. Disease recurrence or death: Without HIPEC: 110 out of 123 patients (89%); With HIPEC: 99 of the 122 patients (81%); (HR: 0.66; 95% CI: 0.50–0.87; *p* = 0.003). 3. Median recurrence-free survival (RFS): Without HIPEC: 10.7 months; With HIPEC: 14.2 months. 4. Median OS: Without HIPEC: 33.9 months; With HIPEC: 45.7 months.
Zhang G (2019)	Meta-analysis including randomized controlled trials and case–control trials	1. Patients with primary stage III/IV ovarian cancers; 2. 13 comparative studies.	Groups: 1. Interval CRS plus HIPEC; 2. Primary CRS plus HIPEC; 3. Without HIPEC.	1. Better outcomes of surgery and HIPEC in patients with primary advanced ovarian cancer (pooled HR for OS: 0.54; 95% CI: 0.45–0.66; pooled HR for PFS: 0.45; 95% CI: 0.32–0.62); 2. Favorable clinical outcome for stage III/IV ovarian cancer with initial diagnosis (HR: 0.64,95% CI: 0.50–0.82, HR: 0.36,95% CI: 0.20–0.65).
Lei Z (2020)	Multicenter retrospective cohort study	1. Patients with stage III primary epithelial ovarian cancers; 2. 584 patients.	1. Closed technique; 2. Circulating heated saline with cisplatin at a dose of 50 mg/m^2^ 3. 43 °C, 60 min.	Median survival time: 1. HIPEC: 49.8 months; 2. Non-HIPEC 34.0 months (HR: 0.63, 95%CI, 0.49–0.82, *p* < 0.001). 3-year overall survival rate: 1. Surgery + HIPEC: 60.3% (95% CI: 55.3–65.0%). 2. Surgery alone: 49.5% (95% CI: 41.0–57.4%) (weighted HR: 0.64; 95% CI: 0.50–0.82; *p* < 0.001).

**Table 3:** A summary of clinical studies investigating HIPEC treatment for recurrent ovarian cancers.

Authors	Study Design	Patients	Treatment	Results
Cascales-Campos (2011)	Descriptive study of outcomes in both primary and recurrent epithelial ovarian cancer	1. Patients previously diagnosed with primary stage IIIc (35 patients) or recurrent ovarian cancer (11 patients) treated using peritonectomy procedures and HIPEC; 2. Total: 46 patients.	A total of 37 patients (80.4%) received systemic chemotherapy (3–18 cycles per patient) before HIPEC and surgery. Regimen dose of HIPEC: 1. Paclitaxel (60 mg/m^2^); 2. Cisplatin (75 mg/m^2^) in taxol-allergic patients 3. 60 min, 42 °C.	1. Median operation time: 380 min (200–540 min); 2. CC-0 (no macroscopic tumor residue at the end of cytoreduction) achieved in 38 patients (82.6%).
Spiliotis (2015)	Prospective randomized phase III study	1. Patients with advanced ovarian cancer (FIGO) IIIc and IV) who experienced disease recurrence after initial treatment with conservative or debulking surgery and systemic chemotherapy; 2. 120 patients.	Groups: HIPEC (group A): 1. CRS was followed by the administration of HIPEC and subsequent systemic chemotherapy; 2. Platinum-sensitive disease (*n* = 34): cisplatin 100 mg/m^2^ + paclitaxel 175 mg/m^2^, 60 min at 42.5 °C; 3. Platinum-resistant disease (*n* = 26): doxorubicin 35 mg/m^2^ + (paclitaxel 175 mg/m^2^ or mitomycin 15 mg/m^2^), 60 min at 42.5 °C. Non-HIPEC (group B): CRS followed by systemic chemotherapy.	Overall mean survival: HIPEC: 26.7 months; Non-HIPEC: 13.4 months (*p* < 0.006). 3-year survival: HIPEC: 75%; Non-HIPEC: 18% (*p* < 0.01).
Zhang G (2019)	Meta-analysis including randomized controlled trials and case–control trials	Patients with recurrent ovarian cancers.	Groups: 1. HIPEC; 2. Without HIPEC.	1. OS: improved for HIPEC group; (HR: 0.45, 95% CI: 0.24–0.83) 2. PFS: no correlation between HIPEC and non-HIPEC group (HR: 0.55, 95% CI: 0.27–1.11).
